# Effects of Herbal Compound (IMOD) on Behavior and Expression of Alzheimer's Disease Related Genes in Streptozotocin-Rat Model of Sporadic Alzheimer’s Disease

**DOI:** 10.15171/apb.2017.060

**Published:** 2017-09-25

**Authors:** Niloofar Bazazzadegan, Marzieh Dehghan Shasaltaneh, Kioomars Saliminejad, Koorosh Kamali, Mehdi Banan, Hamid Reza Khorram Khorshid

**Affiliations:** ^1^Genetics Research Center, University of Social Welfare and Rehabilitation Sciences, Tehran, Iran.; ^2^Laboratory of Neuro-organic Chemistry, Institute of Biochemistry and Biophysics (IBB), University of Tehran, Tehran, Iran.; ^3^Reproductive Biotechnology Research Center, Avicenna Research Institute, ACECR, Tehran, Iran.

**Keywords:** Alzheimer’s disease, Gene expression, Herbal extract, Rat model

## Abstract

***Purpose:*** Sporadic Alzheimer’s disease (AD) accounts for over 95% of cases. Possible mechanisms of AD such as inflammation and oxidative stresses in the brain motivate researchers to follow many therapies which would be effective, especially in the early stages of the disease. IMOD, the herbal extract of R. Canina, T. Vulgare and U. Dioica plant species enriched with selenium, has anti-inflammatory, immunoregulatory and protective effects against oxidative stress.

***Methods:*** In this study three AD-related genes, DAXX, NFκβ and VEGF, were chosen as candidate to investigate the neuroprotective effect of the extract by comparing their expression levels in the hippocampus of rat model of sporadic AD, using qPCR in the herbal-treated and control groups. The therapeutic effects on learning and memory levels were evaluated by Morris Water Maze (MWM) test.

***Results:*** Gene expression results were indicative of significant up-regulation of Vegf in rat’s hippocampus after treatment with the herbal extract comparing to model group (P-value= 0.001). The MWM results showed significant changes in path length and time for finding the hidden platform in all groups during test and the same change in the treated comparing to the control group in memory level.

***Conclusion:*** It could be concluded that the herbal extract may have significant effect on gene expression but not on behavioral level.

## Introduction

Sporadic Alzheimer's disease (AD) is a complex disorder which both genetic and environmental risk factors are involved.^1^ An important event in pathogenesis of AD is aggregation of Aβ peptide in the brain. Most approaches to therapy in AD aimed at preventing aggregation of Aβ peptides.^2^ Sporadic Alzheimer's disease (SAD) is an insulin-resistant brain state. It is proposed that direct injection of streptozotocin (STZ) into rat brain could be used as an AD model (type 3 diabetes).^3,4^ STZ impairs brain glucose and energy metabolism and induces the impairment of learning and memory formation, and moreover lowering of choline acetyl transferase levels in the hippocampus.^3,5^

In AD it is essential to recognize the specific molecular pathways. The expression pattern of genes provides indirect information about function, drug target and cause of a disease. Among various genes related to pathology of SAD, *DAXX*, *NFκβ*, *VEGF* genes with the role in apoptosis, inflammation and angiogenesis represented significant differential expression in Alzheimer human brain.^6^

IMOD (Rose PharMed Co. (Iran)), the herbal extract of *Tanacetum vulgare*, *Rosa canina* and *Urtica dioica* plant species, which has been enriched with selenium, has anti-inflammatory, immunoregulatory and a protective effect against oxidative stress.^7-9^ Several *in vitro* and *in vivo* studies in animal models and human have shown that *Urtica dioica* extracts decreases some inflammatory factors levels. Furthermore, its immunoregulatory properties in inflammatory bowel diseases, immunogenic type-1 diabetes in mouse, sepsis and HIV patients has been evaluated.^10-17^ In this study according to the importance of molecular mechanisms of AD such as inflammation and oxidative stresses in the brain, the neuroprotective effect of this herbal extract was investigated by evaluating the expression levels of the three AD-related genes, *Daxx*, *Nfκβ* and* Vegf*, in the hippocampus of rat model of SAD using qPCR in treated and untreated groups. In addition, the therapeutic effects were checked on behavioral, learning and memory levels.

## Materials and Methods

Thirty seven adult male *Wistar* rats with 250-300 g weight were used in this research. They were kept in cage with enough food and water, in a stable environment at 22°C and 12h light/dark cycle.^18^ Animals were distributed into five groups each containing of six to eight rats. The control group (Eight rats) received no medication and had no surgery. The sham group (Eight rats) received bilateral intracerebroventricular (ICV) injection of aCSF as the vehicle of STZ, the Alzheimer group (Seven rats) with bilateral ICV infusion of STZ (3 mg/kg) five days after surgery as recovery. The ethanol-treated STZ group (Six rats) which received diluted ethanol 86% ( 10 fold dilution) as I.P. as the vehicle of herbal extract,^18^ and the IMOD treated STZ group (Eight rats) received the compound as intrapritoneal (IP) at the dose of 20 mg/kg/day for 21 days after modeling.^19^

All groups of rats were examined for behavioral evaluation using Morris Water Maze (MWM) test.^20^ They subsequently were sacrificed with stereotaxic surgery and all hippocampi were dissected and preserved in RNA protector solution at -20°C.^21^ All procedures were carried out according to the National Institute of Health Guide for the care and use of laboratory animals.^22^

Total RNAs were extracted from all hippocampus tissues using UP100H ultrasonic processor (Germany) and RNeasy Plus Mini Kit (Qiagen, Hilden, Germany) according to the manufacturer's protocol. Purity and integrity of RNAs were specified using Nano-drop spectrophotometer and gel electrophoresis. cDNA synthesis was performed using RevertAid^TM^ First Strand cDNA Synthesis Kit (Fermentas, Thermo Fisher Scientific) according to the manufacturer's protocol.

The relative expression levels of *Daxx, Nfkb* and* Vegf* in rat hippocampus of each group were assessed by SYBR green Real Time PCR (Takara SYBR Master Mix (Shiga, Japan) in an ABI 7500 Real-time PCR system (Applied Biosystem, Foster city, CA, USA). The normalization was done by *Actb* endogenous control.^23,24^ Cycle threshold (Ct) values were used to calculate fold changes in gene expression between groups using REST 2009 software. P-values less than 0.01 for analysis by REST and in other analysis less than 0.05 were considered statistically significant. MWM test data were analyzed by GraphPad Prism 6 software; Kruskal Wallis (Dunn's multiple comparisons test) test was used for three recorded factors (path length, escape latency and swimming speed) in all treated and untreated groups separately during five days.

## Results and Discussion

### Behavioral Results

**Figure 1:**
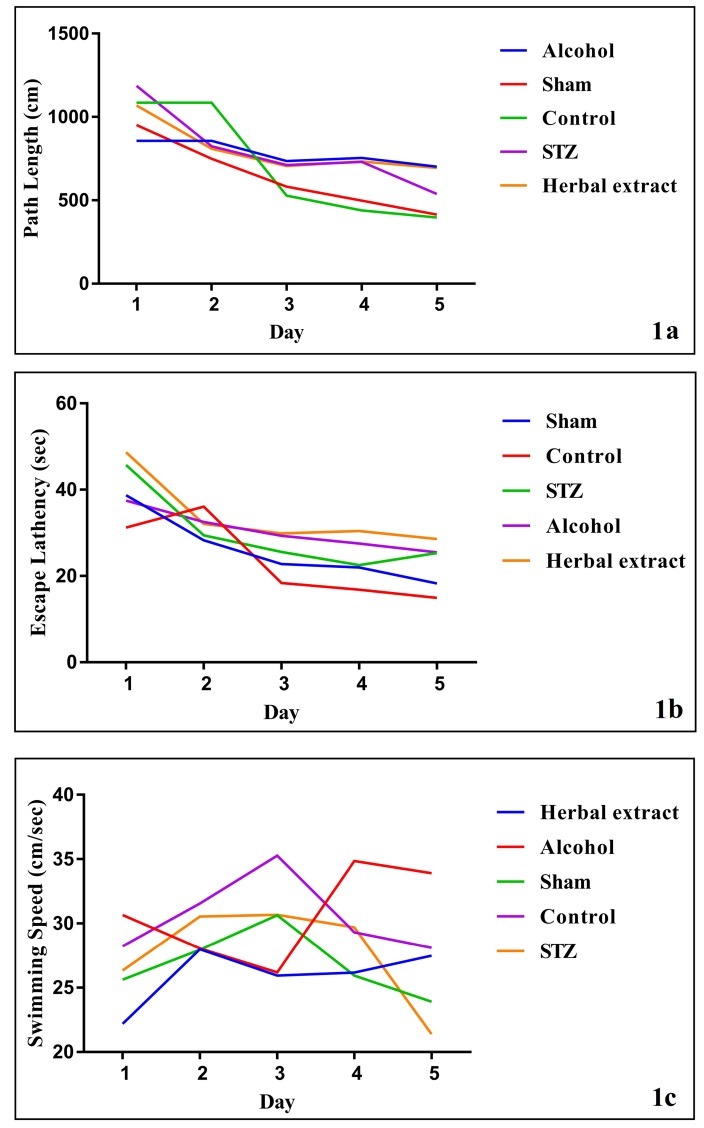
The mean of path length, time for finding hidden platform and swimming speed during five days in all treated and control groups were represented in 1A, 1B and 1C respectively

**Figure 2:**
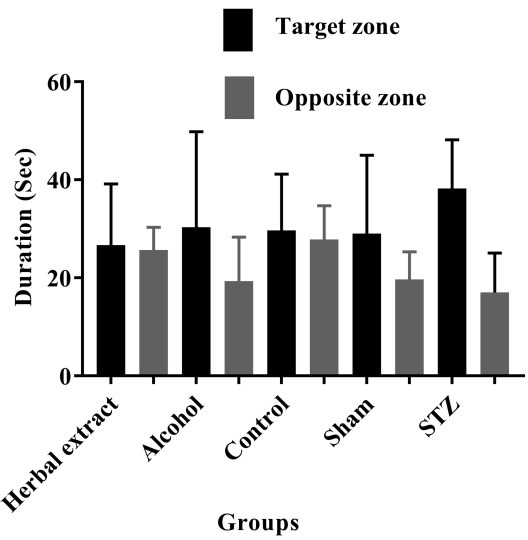
The median with interquartile range of duration time spending in target and opposite zone in the sixth day (four trials) of the test in all treated and control groups

After assessing the learning and memory level changes by Morris Water Maze test, as it is obvious in Figure1, the results showed a significant reduction in swimming distance and time for finding the hidden platform during five days in all groups except alcohol group; however, no significant change was observed in the herbal-treated comparing to the STZ-induced group in path length and escape latency during five days. Probe test indicated no significant change in the Herbal-treated comparing to the control group (Figure 2).

### Gene expression results

**Figure 3:**
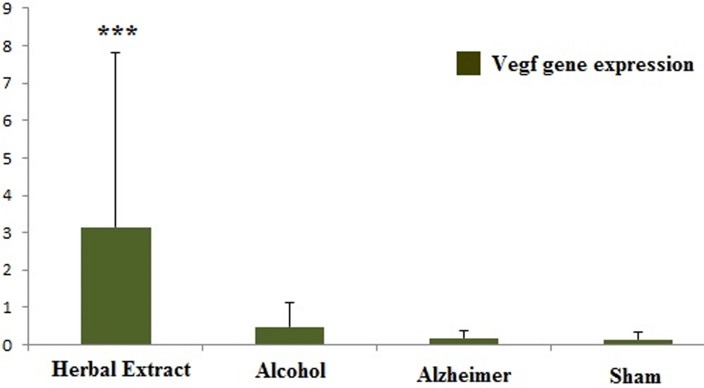
The expression level of *Vegf* gene in all treated and control groups were shown in this figure. * is representative of significant change of gene expression in herbal- treated comparing to Alzheimer group

After evaluating the expression levels of three genes, only *Vegf* gene showed significant (p- value= 0.001) up-regulation in the herbal-treated versus the STZ-induced group (~2.5- fold). In addition, *Vegf* showed a significant down-regulation in the model compared to the control group (P- Value= 0) (Figure 3). Two other genes,* Daxx, Nfkb*, did not show any significant changes in expression level between the herbal-treated and the model group.

In this study, we evaluated the expression of three candidate genes (*Daxx, Nfkb* and* Vegf*) for Alzheimr’s disease in RNA level in rat models of AD. Our results showed that only *Vegf* gene was significantly up-regulated in the herbal-treated compared to the model group. According to the available data, it is postulated reduced *VEGF* expression in AD.^25^ Furthermore, increased levels of VEGF has been reported in the hippocampal cortex of AD patients comparing to normal brain.^26^ Therefore, VEGF levels are controversial in AD patient. In this study, *Vegf* expression showed significant down-regulation in model comparing to control group, but it showed inverse result in treated versus model group. Also, as it has been indicated, *Vegf* showed significant up-regulation in alcohol comparing to model group; whereas, no difference in the herbal comparing to its vehicle (alcohol) group was seen. Thus, alcohol may be an important factor which could be effective on expression level of *Vegf* gene in the herbal-treated group. In behavioral evaluation, as it has been shown in probe test graph (Figure 2), increasing of duration time spent in zone (target) where already the hidden platform has been located there, was observed in STZ group, whereas herbal-treated model showed decreased duration comparing to the model group but the same as control group. Regarding to the recent report by Daneshmand et al.^18^ after evaluating rat treated with this herbal compound comparing to STZ-induced group in expression level and behavioral test, two other AD related genes, *Syp* and *Psen1* showed significant expression change in the herbal-treated group, and also significant increased memory was observed in this group comparing to the other groups.^18^

## Conclusion

In summary, regarding to the both behavioral and gene expression analyses, it would be concluded that this extract may have significant effect on gene expression level related to angiogenesis but, not on clinical levels.

## Acknowledgments

We would like to thank Rose PharMed Co. (Iran) for providing the herbal extract. The study was supported by the University of Social Welfare and Rehabilitation Sciences, Tehran, Iran.

## Ethical Issues

The present study was approved by the Ethical Committee of University of Social Welfare and Rehabilitation Sciences.

## Conflict of Interest

The authors declare no conflict of interest.
